# Prognostic factors and a value of 2009 FIGO staging system in vulvar cancer

**DOI:** 10.1007/s00404-012-2683-x

**Published:** 2012-12-22

**Authors:** Jacek J. Sznurkowski, Tomasz Milczek, Janusz Emerich

**Affiliations:** 1 Department of Oncological Surgery, Medical University of Gdańsk, Gdańsk, Poland; 2 Department of Gynecology, Gynecological Oncology and Gynecological Endocrinology, Medical University of Gdańsk, Gdańsk, Poland

**Keywords:** Vulvar SCC, Prognosis, Survival, Grading, Pathology, FIGO

## Abstract

**Objective:**

In 2009, International Federation of Gynecology and Obstetrics (FIGO) modified staging of vulvar cancer—the prognostic significance of the new classification relative to the prior system as well as to the commonly recognized prognostic factors has not been assessed. The aim of this study was to test prognostic ability of 2009 staging in a cohort of uniformly treated and staged cases with long-term follow-up.

**Methods:**

Pathologic characteristics were obtained by blind review of the original tissue samples. 76 patients who qualified for surgery on the basis of the same criteria, with full clinical history, were included in the study. The histological analyses were performed on 76 and 35 paraffin-embedded tissue samples from primary tumors and lymph nodes, respectively. Survival analyses included the Kaplan–Meier method, log-rank test and Cox proportional hazards model.

**Results:**

Univariate analysis has demonstrated that age (*p* = 0.0170), lymph node metastasis (*p* = 0.0393), tumor grade (*p* = 0.0086) and FIGO1994 stage (*p* = 0.001) were the significant prognostic factors for overall survival. Multivariate analysis has demonstrated that growing age (HR 2.25, 95 % CI 0.79–3.71, *p* = 0.0321), tumor grade (G1 vs. G2 and G3) (HR 1–3.11, 95 % CI 1.6–4.62, *p* = 0.0057) and FIGO1994 stage (HR 1.78, 95 % CI 0.55–3.01, *p* = 0.0061) are independent prognostic factors with respect to overall survival.

**Conclusions:**

The results indicate the prognostic advantage of the 1994 FIGO staging as it has become an independent prognostic factor in contrast to the new FIGO system. This should be tested in future larger cohort studies. Differentiation grade turned out to be a very valuable independent prognostic factor and should be incorporated as a routine component of the histopathologic reports in vulvar cancer.

## Introduction

Vulvar cancer has an incidence of 1–2 per 100,000 women per year and represents 3–5 % of all gynecological malignancies [1–3]. Squamous cell carcinoma (SCC) is a predominating malignancy at this site as it accounts for approximately 85–90 % of vulvar cancers [4, 5]. Acquaintance with the factors influencing prognosis is required and is still a challenge in vulvar cancer because of rarity of this disease.

Most of the knowledge about prognostic factors comes from retrospective analyses of cases with different histological types, collected for a long period, frequently treated surgically in a different way, with pathological data assessed with diverse criteria [6–14].

A new staging system for vulvar cancer was introduced in 2009 by the International Federation of Gynecology and Obstetrics (FIGO) [15] to replace the previous FIGO staging (1988), which was successfully used for over 20 years with only one modification, for stage I, introduced in 1994 [16].

New FIGO staging system has shifted locoregional disease to the lower urethra, vagina or anus to stage II, effectively separating these cases from lymph node positive patients. In addition, larger size, non-metastatic primary lesions were grouped with smaller lesions into stage I. The second substantial change proposed in the 2009 FIGO staging system concerns stage III. This is now reserved for metastatic cases (patients with a tumor of any size with or without extension to the adjacent perineal structures with positive inguino-femoral lymph nodes) and composed of three sub-stages based on the number of lymph nodes involved and extent of involvement of nodes. Staging systems allow accurate prognostication and compare outcomes between centers and countries.

While prognostic significance of previous FIGO stage was evaluated several times [12–14], the new FIGO has not been tested yet.

The aim of this study was to assess prognostic factors in vulvar SCC (including new FIGO stages) cases by analyzing histopathological features obtained by evaluation of tissue samples in cohort planned to surgery consistently with the same algorithm.

## Patients and methods

### Patients and specimens

We studied 110 patients with primary vSCC who had been treated at the Department of Gynaecological Oncology, Medical University of Gdańsk, Poland between January 2002 and December 2006. All patients underwent standard surgical treatment which was not modified by the results of sentinel node procedure. Surgery was classified as follows. Wide local excision (WLE) was performed in case of tumor <2 cm with superficial invasion <1 mm. In case of lateral tumor with invasion of >1 mm, patients were treated with WLE or tailored radical vulvectomy with unilateral inguino-femoral lymphadenectomy. In case of midline tumor, radical vulvectomy in concert with bilateral inguino-femoral lymphadenectomy was performed. Most of lymphadenectomies were performed by separate incisions. Postoperative radiotherapy was given to all patients with positive inguinal lymph nodes, unless there was only one intranodal lymph node metastasis in combination with well-differentiated primary tumor histology.

Clinical data were obtained from the medical records and from the questionnaires designed specially for this study and completed personally by the patients or by their relatives. Histopathological data were obtained by a blind review of all samples retrieved from the archives for the purpose of the study. Tumor type (pT), depth of invasion (measured from the epithelial–dermal junction of the adjacent most superficial dermal papillae to the deepest point of invasion), tumor grade according to the Gynecological Oncology Group (GOG) and lymph nodes status (pN), number and size of lymph nodes metastases, were verified by the same two independent pathologists (without knowledge of the disease outcome). All these patients were staged according to the old and new FIGO systems for vulvar cancer [15, 16]. 34 patients were excluded because of lack of clinical history (*n* = 13), prior anticancer treatment including neoadjuvant radiation or chemotherapy prior to surgery (*n* = 9), incomplete specimens (*n* = 8) and pathology discrepancy (*n* = 4).

Finally, 76 patients with verified histopathological data and full clinical history were included in the study. The histological analyses were performed on 76 and 35 paraffin-embedded tissue samples from the primary tumor and lymph nodes, respectively.

## Methods

The impact of pathological variables, type of the tumor (pT), lymph node status (pN), tumor grade, depth of invasion, FIGO stage (FIGO1994 and FIGO2009), as well as clinical features, age and recurrence, on overall survival was assessed.

### Statistical analysis

In order to determine statistically significant differences between the variables, the Mann–Whitney *U* test was used. Correlations and differences between variables were assessed using the Spearman’s rank correlation coefficient, Chi-square and Fisher tests. The Kaplan–Meier method was used to estimate overall survival, and survival differences were analyzed by the log-rank test and *F* Cox test. *P* values of <0.05 were regarded as significant. For uni- and multivariate analysis, the Cox proportional-hazards regression model was used to explore the impact of individual variables on survival. *P* values of <0.05 were regarded as significant in all of the analyses.

## Results

### Study population

The clinico-pathological data of the patients with primary vulvar SCC and their relation to the course of the disease are summarized in Tables 1 and 2, respectively.

**Table 1:** Clinicopathological characteristics of patients with vulvar SCC

Age, years (median)	69.5 (36–85)
Depth of invasion, mm (median)	7 (0.5–18)
pT status	
pT1a	2 (2.63 %)
pT1b	14 (18.42 %)
pT2	54 (71.05 %)
pT3	5 (6.58 %)
pT4	1 (1.32 %)
pN status	
pN0	24 (31.58 %)
pN1	23 (30.26 %)
pN2	12 (15.79 %)
pNX	17 (22.37 %)
Histologic grade	
G1	27 (35.53 %)
G2	29 (38.15 %)
G3	20 (26.32 %)
FIGO2009	
Ia	2 (2.63 %)
Ib	37 (48.68 %)
II	2 (2.63 %)
IIIa	7 (9.21 %)
IIIb	17 (22.37 %)
IIIc	7 (9.21 %)
IVa	4 (5.26 %)
FIGO1994	
Ia	2 (2.63 %)
Ib	12 (15.79 %)
II	25 (32.89 %)
III	24 (31.58 %)
IVa	13 (17.11 %)

vSCC patients (*n* = 76) follow up: median = 51.23 months (range 6.33–135.5)

**Table 2:** Clinical and histopathological characteristics of the vSCC patients related to the course of the disease

Clinical and histopathological features	No recurrence	Local recurrence	Groin recurrence
	*n* = 61 (80.26 %)	*n* = 12 (15.79 %)	*n* = 3 (3.95 %)
Age, years, median (range)	68 (36–85)	73 (55–82)	75.3 (63–85)
Depth of invasion (mm), median (range)	6.58 (0.5–14.0)	7.82 (2.0–18.0)	9 (6.0–10.0)
Grade G1	24 (39.34 %)	2 (16.67 %)	1 (33.33 %)
Grade G2	23 (37.71 %)	6 (50 %)	0 (0 %)
Grade G3	14 (22.951)	4 (33.33 %)	2 (66.67 %)
Number of positive inguinofemoral lymph nodes	1.24 (SD 1.91)	6.16 (SD 6.73)	2.67 (SD 3.06)
FIGO2009			
Ia	2	0	0
Ib	33	3	1
II	1	1	0
IIIa	7	0	0
IIIb	11	5	1
IIIc	5	1	1
IVa	2	2	0
FIGO1994			
Ia	2	0	0
Ib	9	2	1
II	24	1	0
III	19	4	1
IV	7	5	1

Briefly, the median age of the patients was 69.5 years (range 36–85), the median duration of follow-up was 51.23 months (range 6.33–135.5), the median overall survival was 41.16 months (range 1.7–98.43). 5 years disease-free survival (DFS) was 65 %. Recurrence was observed in 15 patients (15/76, 19.74 %). 12 had local recurrence (12/76, 15.79 %) and 3 revealed recurrence in the groin (3/76, 3.95 %).

**Fig. 1:**
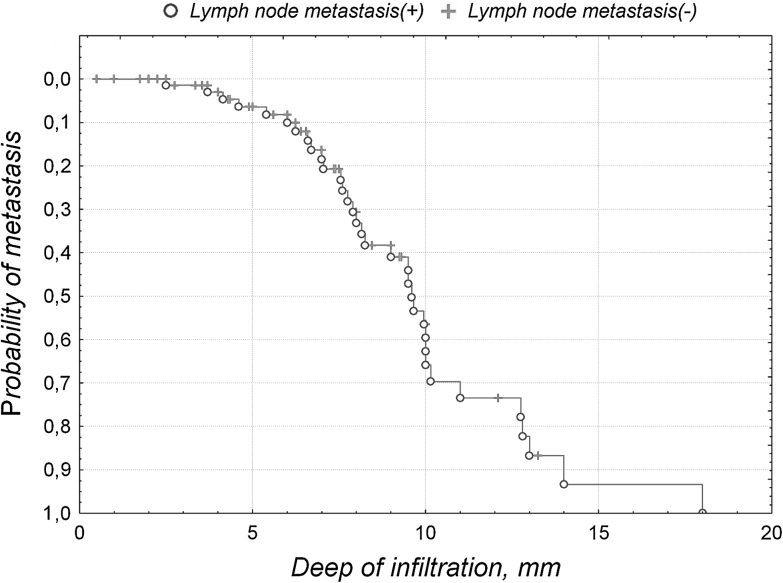
The probability of inguino-femoral lymph node metastasis in relation to depth of invasion of primary tumor

Depth of invasion in metastatic (median 8.2 mm) and non-metastatic cases (median 5.6 mm) was significantly different (*U*–MW test, *p* = 0.00006). The probability of inguino-femoral lymph node metastasis increased with depth of invasion of primary tumor (Fig. 1).

The inverse correlation between histologic tumor grade (GOG) and type of the tumor (pT) (RSpearman = −0.27, *p* = 0.017) and lymph node status (RSpearman = −0.24, *p* = 0.037) was observed.

### Prognostic value of clinicopathological variables

#### pT and pN status (according to TNM system)

**Fig. 2:**
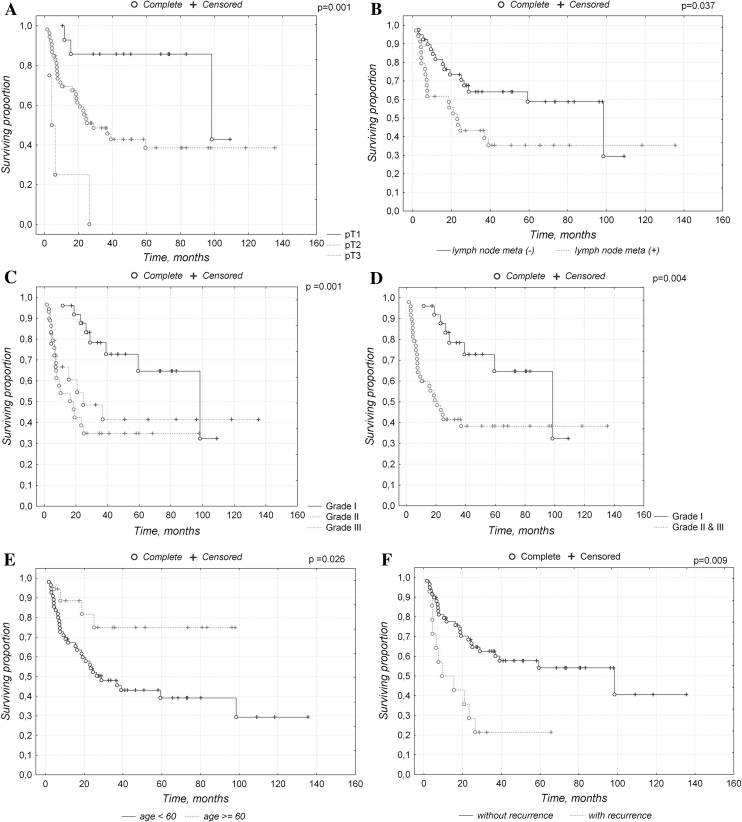
Kaplan–Meier survival curves for overall survival of patients by: tumor type (**a**), nodal status (**b**), tumor grade G1/G2/G3 (**c**) and by differentiated (G1)/undifferentiated tumors (G2 + G3) (**d**) in vSCC patients, age (below/over 60 years) (**e**), recurrence (**f**)

Type of the tumor (pT: T1, T2, T3, T4) has significant impact on overall survival (*p* = 0.001) (Fig. 2a) as well as nodal status (pN: N0, N1, N2) (*p* = 0.037) (Fig. 2b).

#### Histologic tumor grade

We found significant differences in overall survival between patients with different histologic tumor grades (divided in accordance with three-tier grading scheme: G1/G2/G3) (Fig. 2c) as well as between cases with well differentiated (differentiation grade 1) and poorly differentiated tumors (differentiation grades II–III) (Fig. 2d).

#### Depth of invasion

We did not manage to find any borderline depth of invasion with significant impact on overall survival (*p* = 0.736).

#### FIGO stage

The stage distribution according to the 1996 FIGO staging system was stage IA: 2 (2.63 %), stage IB: 12 (15.79 %), stage II: 25 (32.89 %), stage III: 24 (31.58 %) and stage IVA: 13 (17.11 %). The cumulative 5-year survival under the old system was stage I: 83 %, stage II: 47 %, stage III: 41 % and stage IV: 23 % (*p* = 0.00253).

**Fig. 3:**
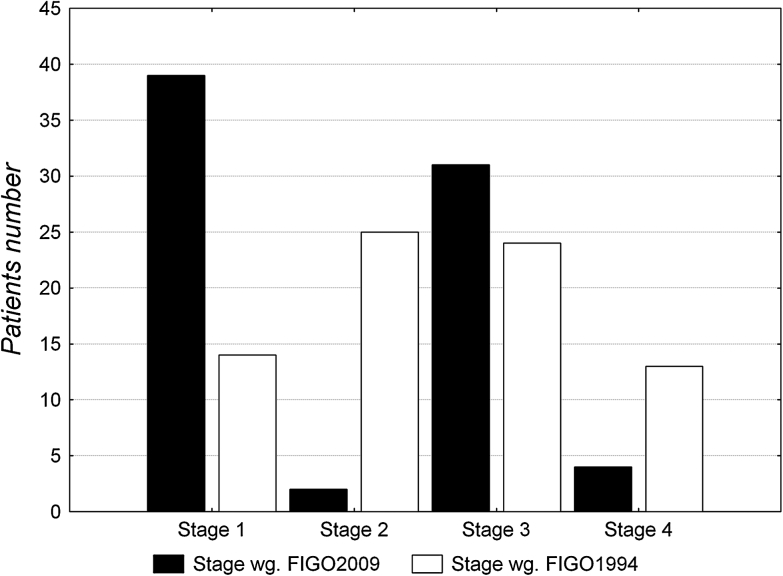
Distribution of patients in stages of FIGO1994 and 2009

The distribution changed under the 2009 FIGO system to stage IA: 2 (2.63 %), stage IB: 37 (48.68 %), stage II: 2 (2.63 %), stage III: 31 (40.79 %) and stage IVA: 4 (5.26 %). The cumulative 5-year survival also changed to stage I: 61 %, stage II: 0 % and stage III: 36 % (*p* = 0.11689). For stage IVA, the period of observation was not long enough to establish 5-year survival. The stage distribution in both FIGO staging systems is presented in Fig. 3.

Twenty-five patients with stage II, 2 patients with stage III and 10 patients with stage IVA classified in accordance to previous 1994 FIGO system were down-staged in the new FIGO classification to stage IB, II and III, respectively. One patient with 1994 FIGO stage III was up-staged to 2009 FIGO stage IVA.

#### Age

Patients older than 60 years had significantly worse prognosis (*p* = 0.026) (Fig. 2e).

#### Recurrence

Recurrence was correlated with decreased overall survival (*p* = 0.009) (Fig. 2f).

### Univariate and multivariate analyses of prognostic variables in vulvar SCC patients

Univariate analysis has demonstrated that age (*p* = 0.0170), lymph node metastasis (*p* = 0.0393), tumor grade (G1 vs. G2 and G3) (*p* = 0.0086) and FIGO1994 (*p* = 0.001) were the significant prognostic factors for overall survival (Table 3).

**Table 3:** Univariate analysis of prognostic variables in vulvar SCC patients

Variable	Category	Overall survival		*p*
		Hazard ratio	95 % Confidence interval	
Age	Continuous	1.04	0.02–2.06	0.0170
Metastases	Negative	1	0.61–3.41	0.0393
	Positive	2.01		
Histologic grade	Low (I)	1	1.38–4.38	0.0086
	High (II and III)	2.88		
FIGO2009	I, II, III, IV	1.41	0.24–2.58	0.03
FIGO1994	I, II, III, IV	1.80	0.6–3	0.001

Multivariate analysis has confirmed that growing age (HR 2.25, 95 % CI 0.79–3.71, *p* = 0.0321), tumor grade (G1 vs. G2 and G3) (HR 1–3.11, 95 % CI 1.6–4.62, *p* = 0.0057) and FIGO1994 (HR 1.78, 95 % CI 0.55–3.01, *p* = 0.0061) have been found to be independent prognostic factors in respect to overall survival (Table 4).

**Table 4:** Multivariate analysis of prognostic variables in vulvar SCC patients

Variable	Category	Overall survival		*p*
		Hazard ratio	95 % Confidence interval	
Age	Continuous	2.25	0.79–3.71	0.0321
Histologic grade	Low (I)	1	1.6–4.62	0.0057
	High (II and III)	3.11		
FIGO1994	I, II, III, IV	1.78	0.55–3.01	0.0061

## Discussion

All histopathological data were obtained by evaluation of original tissue samples in cohort planned for surgery consistently with the same algorithm.

We believe that blind review of all samples provided by two independent pathologists improved the value of the results. Several parameters were not incorporated in the available archival diagnostic reports (e.g. differentiation grade, size and number of lymph node metastasis, presence of extracapsular spread), and depth of invasion was previously assessed using diverse criteria. The importance of proper measurement of the depth of invasion in vSCC was recently indicated by Yoder et al. [17].

The utilized algorithm for type and extent of primary surgery was consistent with widely accepted and even obligatory rules based on available evidence between 2002 and 2006 [18–21]. Suspicious pelvic lymph nodes were not excised in the analyzed cohort. Postoperative radiotherapy to the groin and pelvis was given to all patients with positive inguinal lymph nodes, unless there was only one intranodal lymph node metastasis in combination with a well-differentiated primary tumor.

Most of the available reports on prognostic factors in vSCC [13, 14] have included the analyses of cases treated with pelvic lymphadenectomy while it has been proven that such modality has negative influence on overall survival. Advantage of radiation to the pelvis in patients with positive inguinal lymph nodes as well as clinically suspected or fixed ulcerated groin nodes was confirmed in prospective randomized trials [22, 23]. Therefore, survival analyses provided in appropriately treated cohort (up to date) serve as more reliable results.

Study group was observed for long enough (median 51.23 months) to reveal all potential recurrences [8–10, 24–27]. Our patients had a 5-year DFS of 66.5 % and a recurrence rate of 20 % with 35 % patients in stage III and IV. While the long-term survival was comparable to those reported in the literature [8–10, 24–27], we notified lower recurrence rate than others [24, 25].

While depth of invasion in metastatic (median 8.2 mm) and non-metastatic cases (median 5.6 mm) was significantly different and the probability of inguino-femoral lymph node metastasis increased with depth of invasion of primary tumor, we were not able to find any borderline depth of invasion with significant impact on overall survival in our group of patients. Nicoletto et al. [14] found stromal invasion of over 9 mm to be one of the most dominant predictor for relapsed free survival in vSCC.

Depth of invasion could be evaluated with at least three various measurement techniques, depending on whether the tumor surface, or the ulcer base was chosen as the starting point [28] even in one institution. To standardize this parameter, we utilized only one, most recommended technique (measuring from the most superficial dermal papilla adjacent to the tumor to the deepest focus of invasion). This as well as smaller number of cases in Italian study could explain discrepancies between the two analyses.

Thirty-five patients (35/76, 46 %) were down-staged and one case (1/76, 1 %) was up-staged using the 2009 FIGO system. The results of the overall survival according to both FIGO systems indicated that the new staging stratified survival between stages less effectively than the old FIGO system.

To the best of our knowledge, there were no previous comparative analyses of the old and revised staging systems in vulvar cancer; therefore, we had no adequate source of reference to compare our results.

Conducted multivariate analysis has shown that the growing age, histologic tumor grade and FIGO1994 stage are the independent prognostic factors for overall survival in analyzed group of vSCC patients.

This study together with several previous examinations has demonstrated that differentiation grade plays an important role in the aggressiveness of a tumor and has a considerable impact on survival [9, 10, 13, 14, 17, 27].

The results emphasize the prognostic advantage of the 1994 FIGO staging system as it has become an independent prognostic factor in contrast to the new FIGO system.

The single paper published in 2010 reported that the proposed modifications were successful and the new FIGO staging system provides a better reflection of prognosis [29], but it was followed by the letter to the editor suggesting inverse conclusion [30]. Lack of prognostic significance of 2009 FIGO staging system indicated in current study should be tested in future larger cohort studies.

The role of a pathologist is to provide clinicians with a diagnosis and with as much prognostic information as possible when examining biopsy material [28]. While the data do seem to support that there is an important prognostic role for histologic grade for vSCC [9, 10, 13, 14, 17, 27], most pathologists have not yet incorporated this parameter into common practice schemes. Towards this end, it may be prudent to consider incorporating comments about histologic tumor grade as a routine component of the diagnostic reports of this malignancy.

This study has the traditional weaknesses of a retrospective design and results obviously represent a small cohort. Its strengths include uniformly treated cohort without the effect of treatment evolution over long periods of time and the ability to review pathologic slides to correctly assign newer sub-staging criteria as well as other pathological features.

## Conclusion

Lack of prognostic significance of 2009 FIGO staging system should be tested in future larger cohort studies. Differentiation grade is a very valuable independent prognostic factor and should be incorporated into routine histopathologic reports in vSCC.
